# Posttraumatic stress disorder according to DSM-5 and DSM-IV diagnostic criteria: a comparison in a sample of Congolese ex-combatants

**DOI:** 10.3402/ejpt.v6.24981

**Published:** 2015-02-25

**Authors:** Susanne Schaal, Anke Koebach, Harald Hinkel, Thomas Elbert

**Affiliations:** 1 Department of Psychology, University of Ulm, Ulm, Germany; 2 Department of Psychology, University of Konstanz, Konstanz, Germany; 3 World Bank Kigali, Kigali, Rwanda

**Keywords:** PTSD, DSM-5, DSM-IV, Congo, ex-combatants, violence

## Abstract

**Background:**

Compared to DSM-IV, the criteria for diagnosing posttraumatic stress disorder (PTSD) have been modified in DSM-5.

**Objective:**

The first aim of this study was to examine how these modifications impact rates of PTSD in a sample of Congolese ex-combatants. The second goal of this study was to investigate whether PTSD symptoms were associated with perpetrator-related acts or victim-related traumatic events.

**Method:**

Ninety-five male ex-combatants in the eastern Democratic Republic of Congo were interviewed. Both the DSM-IV and the DSM-5 PTSD symptom criteria were assessed.

**Results:**

The DSM-5 symptom criteria yielded a PTSD rate of 50% (*n*=47), whereas the DSM-IV symptom criteria were met by 44% (*n=*42). If the DSM-5 would be set as the current “gold standard,” then DSM-IV would have produced more false negatives (8%) than false positives (3%). A minority of participants (19%, *n*=18) indicated an event during which they were involved as a perpetrator as their most stressful event. Results of a regression analysis (*R* ^2^=0.40) showed that, after accounting for the number of types of traumatic events, perpetrated violent acts were not associated with the symptom severity of PTSD.

**Conclusions:**

The findings demonstrate that more diagnostic cases were produced with the DSM-5 diagnostic rules than were dropped resulting in an increase in PTSD rates compared to the DSM-IV system. The missing association between PTSD symptoms and perpetrated violent acts might be explained by a potential fascinating and excited perception of these acts.

The Diagnostic and Statistical Manual for Mental Disorders, Fifth Edition (DSM-5; American Psychiatric Association, 2013), introduced several changes of the diagnostic criteria for a posttraumatic stress disorder (PTSD) compared to DSM-IV (American Psychiatric Association, 2000). Major modifications include: 1) the elimination of criterion A2 (the subjective reaction to the traumatic event), 2) the revision of the description of the DSM-IV symptoms, 3) the addition of three new symptoms, and 4) the replacement of the DSM-IV three-symptom cluster structure (B, C, D) with a four-symptom cluster structure (B, C, D, E). Table 1 provides an overview of the DSM-IV and DSM-5 PTSD symptoms.

**Table 1:** Frequency of Symptom Endorsement according to DSM-IV and DSM-5 symptoms of PTSD (rated ≥1 at the PSS-I, Foa & Tolin, 2000)

DSM-IV items	%	*n*	DSM-5 items	%	*n*
Reexperiencing (B)			Reexperiencing (B)		
B1: Intrusions	83.2	79	B1: Intrusions	83.2	79
B2: Nightmares	53.7	51	B2: Nightmares	53.7	51
B3: Flashbacks	65.3	62	B3: Flashbacks	65.3	62
B4: Emotional Reactivity	77.9	74	B4: Emotional Reactivity	77.9	74
B5: Physical Reactivity	76.8	73	B5: Physical Reactivity	76.8	73
Avoidance (C)			Avoidance (C)		
C1: Avoid thoughts/feelings	65.3	62	C1: Avoid thoughts/feelings	65.3	62
C2: Avoid places/activities	61.1	58	C2: Avoid places/activities/objects/situations/conversation^a^	69.5	66
C3: Amnesia	14.7	14	Negative alterations in mood/cognitions (D)		
C4: Loss of interest	44.2	42	D1: Amnesia	14.7	14
C5: Social detachment	33.7	32	D2: Negative beliefs^a^	51.6	49
C6: Psychological numbing	23.2	22	D3: Distorted blame^a^	66.3	63
C7: Foreshortened future	56.8	54	D4: Negative emotional state^a^	53.7	51
			D5: Loss of interest	44.2	42
			D6: Social detachment	33.7	32
			D7: Low positive emotions^a^	21.1	20
Hyperarousal (D)			Hyperarousal (E)		
D1: Difficulty sleeping	62.1	59	E1: Aggression/irritability/anger^a^	27.4	26
D2: Irritability/anger outbursts	41.1	39	E2: Reckless/self-destructive^a^	22.1	21
D3: Difficulty concentrating	67.4	64	E3: Hypervigilance	37.9	36
D4: Hypervigilance	37.9	36	E4: Exaggerated startle response	38.9	37
D5: Exaggerated startle response	38.9	37	E5: Difficulty concentrating	67.4	64
			E6: Difficulty sleeping	62.1	59

a Symptoms not included in the DSM-IV.

To date, few studies have examined the impact of the introduced changes on rates of PTSD diagnosis (e.g., Elhai et al., 2012; Miller et al., 2013). It therefore remains largely unclear how the new DSM-5 PTSD criteria will affect the likelihood of a diagnosis and thus the prevalence estimates of PTSD. The goal of the present study was to compare the DSM-IV and the DSM-5 PTSD diagnostic algorithms in a sample of Congolese ex-combatants.

For more than two decades, the eastern Democratic Republic of Congo (DRC) has been affected by an ongoing cycle of war, violence, and insecurity. Mutual fighting amongst various local armed groups and forces persists within the eastern part of the DRC (Romkema, 2007). By 2012, the security and political situation in the eastern DRC considerably deteriorated and violence increased. Heavy fighting between the Congolese army and a former rebel group, the M23 rebels, erupted. The detrimental mental health effects of exposure to violence are not only common for civilian victims, but also for soldiers and combatants. Combatants have often been perpetrators, although at the same time they have also been victims, as they also experience massive forms of violence and often fear for their lives. Both may have mental health consequences and may lead to PTSD. Some authors have argued that the engagement in intense violence against others may be considered as a potentially traumatic event, which may also cause trauma-related symptoms (MacNair, 2002; Staub, 2006). In particular, the killing of someone during combat has been described as a risk factor for the development of PTSD (Maguen et al., 2009, 2011, 2013; Van Winkle & Safer, 2011). According to Pollock (1999), the perpetration of violent acts may be traumatizing to a perpetrator if the episodes of aggression violates the offender's schema-based assumptions. However, most studies have not investigated whether the trauma related symptoms of those who engaged in violence were related to self-perpetrated or self-experienced events. A recent study of Rwandan perpetrators found that only a few indicated a perpetrated event as the index trauma to which their PTSD symptomatology referred (Schaal, Heim, & Elbert, 2014).

The main goals of the present study were to compare rates of PTSD according to the DSM-IV and DSM-5 symptom criteria in a sample of Congolese ex-combatants and to examine whether PTSD symptom severity is associated with perpetrated violent acts while controlling for general traumatic events. We hypothesized that, after accounting for traumatic event types, perpetrated violent acts would be associated with more severe symptoms of PTSD.

## Method

### Participants and procedure

Eligible participants were ex-combatants who were at the demobilization camp of the United Nations in Goma, DRC. The demobilization camp is a transition camp for all combatants who leave any armed group in the province of North Kivu and report to the United Nations. Participants stay only between 20 and 72 hr in the demobilization camp before being sent to other places.

In total, 95 male Congolese ex-combatants were interviewed. All men approached agreed to participate and completed the diagnostic interview. The participants’ mean age was 24.36 years (SD=6.46, range: 16–46 years). They had served as combatants for an average of 4.77 years (SD=4.62, range: 0.08–24.08 years) and indicated that they had fought for an average of 1.82 armed groups during their life (SD=1.01, range: 1–5). Sixty-five point three percent (*n*=62) reported that they had been forcibly recruited at least once, whereas 57.9% (*n*=55) reported having voluntarily joined an armed group at least once.

The study was conducted in Goma in the province of North Kivu in the eastern DRC between February and April 2013. It was approved by the University of Konstanz Ethical Review Board and the United Nations’ mission in the DRC (MONUSCO). All ex-combatants who arrived at the demobilization camp during the following assessment periods were interviewed: 2–11 February, 27 February–13 March, and 26 March–5 April. After their arrival at the camp, participants were approached and fully informed of the study's procedure and aims, including voluntary participation. All ex-combatants approached agreed to participate and provided us their signed written informed consent. Diagnostic interviews were carried out by a group of local interviewers (four local psychology students from the University of Goma and one translator). All local interviewers had already received extensive training in conducting structured diagnostic interviews during summer 2012 and had already conducted diagnostic interviews in previous investigations. The training covered basic theoretical concepts as well as sensitive and empathic interviewing techniques. Before data collection, the interviewers received follow-up training that lasted 7 days and focused on learning the DSM-5 PTSD diagnostic criteria. The various structured interviews and the questions referring to the DSM-5 symptom criteria were translated into Kiswahili and blind-reverse translated by independent groups of translators. During the whole phase of data collection, interviewers were closely supervised by clinical experts and received extensive feedback. Interviews lasted between 1.5 and 2.5 hr and were carried out individually at a private place at the demobilization camp. No financial compensation was given to the participants.

### Measures

Interviewers obtained socio-demographic data from each respondent. The interviewers measured potentially traumatic events using a 22-item event checklist, which assessed the lifetime exposure to different potentially traumatic event types (traumatic event list; possible range: 0–22; Cronbach's *α*=0.70; possible range of experienced traumatic event types: 0–12; possible range of witnessed traumatic event types: 0–10). This checklist was a version of a previously published checklist (Neuner et al., 2004) that we adapted to fit the Congolese cultural context. The types of lifetime perpetrated violent acts were assessed using a 21-item checklist (perpetrated violence list; possible range: 0–21; Cronbach's *α*=0.88). Perpetrated violent acts included the commission of any act of violence (offensive and defensive acts of aggression) independently of the context in which they took place. Participants were asked to indicate the most stressful event they had ever experienced (from the traumatic event list or the perpetrated violence list), to which the subsequent rating of PTSD symptoms referred.

The DSM-IV PTSD diagnostic status and symptom severity were determined using the PTSD Symptom Scale Interview (PSS-I, Foa & Tolin, 2000). The PSS-I assesses the 17 DSM-IV symptom criteria for PTSD and refers to symptoms experienced in the previous month. Each of the items was answered on a four-point scale ranging from 0 (not at all/only once) to 3 (five or more times per week/almost always). A DSM-IV PTSD severity score (possible score range 0–51; Cronbach's *α*=0.89) was computed by adding all symptom scores.

The DSM-5 PTSD diagnostic status and symptom severity were assessed using the 20 DSM-5 items, which were scored on a four-point scale ranging from 0 (not at all/only once) to 3 (five or more times per week/almost always). A DSM-5 PTSD severity score (possible scores range 0–60; Cronbach's *α*=0.90) was computed by adding all symptom scores. To avoid any repetition of the questions, we have asked items which occur in both diagnostic systems (DSM-IV and DSM-5) only once. The new symptoms of DSM-5 PTSD that did not overlap with the DSM-IV items (Table 1) were integrated into the PSS-I (Foa & Tolin, 2000) and added to the respective symptom cluster. The additional items were created using phrasing similar to that found in the criteria. Table 1 indicates the DSM-5 symptoms which had not been included in the DSM-IV. The exact wording of the new DSM-5 items can be seen in Table 2. Consequently, all participants were evaluated for the presence of DSM-IV-based and DSM-5-based PTSD diagnostic criteria. The diagnostic instruments were administered as clinical interviews.

**Table 2:** Exact wording of the new DSM-5 items integrated into the PSS-I (Foa & Tolin, 2000)

Item	Wording
C2	Have you persistently been making efforts to avoid objects, situations or conversations that arouse distressing memories, thoughts or feelings about, or that are closely associated with the traumatic event?
D2	Have you had strong negative beliefs or expectations about yourself, others, or the world? (e.g., “I am bad,” “No one can be trusted,” “The world is completely dangerous”, “My whole nervous system is permanently ruined”)
D3	Have you blamed yourself or others about the traumatic event, its cause or consequences? (do not include the aggressor)
D4	Have you had any strong negative feelings (e.g., fear, horror, anger, guilt, or shame)?
D7	Have you felt difficulty to experience positive feelings? (e.g., unable to have loving or happy feelings)
E1	Have you shown irritable or aggressive behavior? (expressed verbally or physically towards people or objects)
E2	Have you shown reckless or self-destructive behavior (doing things that might have caused you harm)?

### Data analysis

The presented descriptive data are expressed as frequencies (%), mean scores, and standard deviations. Pearson's chi-square analyses are used to analyze between-group differences. We report Cronbach's *α* as measure for internal consistency. Classical test theory requests that scales should have a high degree of internal consistency as moderately evidenced by the observed values. It should also be noted that Cronbach's *α* overstates the reliability, however the PDS, for example, may be composed of sets of measures that may not necessarily be correlated. To investigate the association between the DSM-5 PTSD symptom severity and experienced event types, hierarchical linear regression analysis was calculated for the DSM-5 PTSD severity score. The number of traumatic event types (experienced and witnessed) was entered in step 1, followed by the number of types of perpetrated violent acts in step 2. The regression model fulfilled all necessary quality criteria for linear regression analyses. The residuals did not significantly deviate from normality, linearity, or homoscedasticity. No univariate outliers could be identified. The maximum variance inflation factor did not exceed 2.03. Hence, we do not need to take multicollinearity into account. Data analysis was conducted using version 21 of the SPSS software. Listwise deletion was applied to deal with missing data. The reported statistical tests were two-tailed.

## Results

### Trauma exposure and perpetrated violent acts

All participants had been exposed to at least one traumatic event (A1, DSM-IV and DSM-5) and all participants reported a subjectively felt response involving intense fear, helplessness, or horror (A2, DSM-IV). The five most prevalent types of potential traumatic events were “witnessing dead bodies” (97.9%, *n*=93), “witnessing physical assault” and “witnessing assault with a weapon” (95.8%, *n*=91, respectively), “assault with a weapon” and “witnessing a killing” (91.6%, *n*=87, respectively); the five most often reported types of perpetrated violent acts included “defense in a fight” and “hitting back when being attacked” (96.8%, *n*=92, respectively), “killing someone” (92.6%, *n*=88), “making another person bleed” (87.4%, *n*=83), and “physical assault with a weapon” (86.3%, *n*=82).

### Frequency of symptom endorsement and rates of PTSD

Table 1 displays the rate of endorsement of each of the DSM-IV and DSM-5 symptoms. The most frequently reported DSM-IV and DSM-5 symptoms for both diagnostic systems included symptoms from the B-symptom cluster of re-experiencing. Table 3 lists the frequency of endorsement of each of the DSM-IV and DSM-5 diagnostic criteria of PTSD.

**Table 3:** Frequency of PTSD Symptom Criteria Endorsement according to DSM-IV and DSM-5

DSM-IV symptom criteria				DSM-5 symptom criteria			

	%	*n*	Cronbach's *α*		%	*n*	Cronbach's *α*
Criterion A	100.0	95		Criterion A	100.0	95	
Criterion B	86.3	82	0.85	Criterion B	86.3	82	0.85
Criterion C	55.8	53	0.74	Criterion C	80.0	76	0.88
Criterion D	65.3	62	0.84	Criterion D	75.8	72	0.64
Criterion E	86.3	82		Criterion E	67.4	64	0.81
Criterion F	85.3	81		Criterion F	86.3	82	
				Criterion G	85.3	81	
				Criterion H	93.7	89	
PTSD diagnosis	44.2	42		PTSD diagnosis	49.5	47	

The percentages of participants who met each individual DSM-5 symptom criterion were as follows: 86.3% (*n*=82) criterion B, 80.0% (*n*=76) criterion C, 75.8% (*n*=72) criterion D, and 67.4% (*n*=64) criterion E. The mean DSM-IV PTSD sum score was *M*=15.42 (SD=9.60; range: 0–40); the mean DSM-5 PTSD sum score was *M*=17.72 (SD=10.87; range: 0–48).

**Fig. 1:**
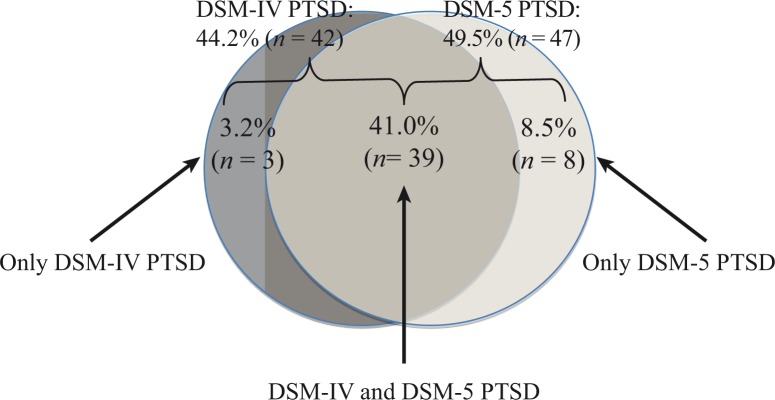
PTSD diagnosis according to DSM-IV and DSM-5 symptom criteria.

Forty-four point two percent (*n*=42) of the total sample met DSM-IV PTSD diagnostic criteria, and 49.5% (*n*=47) fulfilled criteria for a DSM-5 PTSD diagnosis. Forty-one percent (*n*=39) fulfilled PTSD criteria according to both diagnostic algorithms; 3.2% (*n*=3) only according to DSM-IV, and 8.5% (*n*=8) only according to DSM-5 (Fig. 1). The three participants who fulfilled diagnostic criteria according to DSM-IV (but not DSM-5) were omitted because two participants no longer fulfilled the DSM-5 D-criterion and one person did not meet the DSM-5 H-criterion. The eight participants who fulfilled diagnostic criteria according to DSM-5 (but not DSM-IV) were newly added because they did not meet the DSM-IV C-criterion (where three symptoms were required), but now fulfilled the DSM-5 C-criterion (where only one symptom is required). The majority (72.3%, *n*=34) of participants who met DSM-5 PTSD criteria also displayed dissociative symptoms (depersonalization: 70.2%, *n*=33; derealization: 72.3%, *n*=34). Of those with DSM-IV PTSD, 92.9% (*n*=39) met DSM-5 PTSD; of those who met criteria for a current DSM-5 diagnosis, 83.0% (*n*=39) also fulfilled DSM-IV diagnostic criteria. A minority of participants (8.4%, *n*=8) met PTSD criteria according to the DSM-5 algorithm but not according to the DSM-IV algorithm. In other words, if the DSM-5 would be set as the current “gold standard,” then DSM-IV would have produced 8.4% false negatives and produced 3.2% false positives.

### Association between event type and PTSD

Most of the ex-combatants indicated “witnessing a killing” (22.1%, *n*=21) as the most stressful event they had ever experienced, followed by “assault with a weapon” (20.0%, *n*=19), “witnessing a massacre” (14.7%, *n*=14), and “killing someone” (10.5%, *n*=10) (categories reported by less than 10% of participants have been omitted). From the total sample, the majority of the interviewed (81.1%, *n*=77) indicated an event from the traumatic event list as the most stressful event they had ever experienced; 18.9% (*n*=18) indicated an event during which they themselves had perpetrated violent acts as their most frightening event (perpetrated violence list). Those participants who indicated an event from the perpetrated violence list as their most stressful event that they had ever experienced displayed significant more severe DSM-5 PTSD symptoms compared to those who described an event from the traumatic event list; *M*=24.22 (SD=11.39) versus *M*=16.19 (SD=10.23), respectively, *t*(95)=−2.93, *p*=0.004. Moreover, they fulfilled significantly more frequently the diagnostic criteria for DSM-5 PTSD than ex-combatants who reported an event from the traumatic event list as their most stressful event; 72.2% (*n*=13) versus 44.2% (*n*=34), respectively, *χ* ^2^(1, *N*=95)=4.60, *p*=0.032.

As we assumed a potential overlap between the different predictor variables of PTSD symptom severity, we conducted a hierarchical linear regression analysis. Results are presented in Table 4. The variables of number of experienced event types and number of witnessed traumatic event types were entered in step 1, followed by the variable of number of types of perpetrated violent acts in step 2. Both traumatic event types (experienced and witnessed traumatic events) were significantly positively associated with the DSM-5 PTSD severity score. The explained variance of this model was 38.3%. Step 2 revealed that the number of traumatic event types remained significant, whereas the number of types of perpetrated violent acts did not significantly contribute to the prediction of the severity of PTSD symptoms. This final model accounted for 39.8% of the variance in explaining severity of PTSD symptoms.

**Table 4:** Hierarchical multiple regression analyses with DSM-5 PTSD severity score as the dependent variable (*N*=95)

Variable/model	*R* ^2^	*f* ^2^	*df*	*F*	*B*	*SE B*	*β*
Step 1	0.38	0.61	2	28.54***			
Traumatic event types experienced					2.08	0.57	0.36***
Traumatic event types witnessed					2.30	0.68	0.34***
Step 2	0.40	0.67	3	20.09***			
Traumatic event types experienced					1.74	0.61	0.30**
Traumatic event types witnessed					1.73	0.77	0.25*
Types of perpetrated violent acts					0.43	0.28	0.18 ns

*Note*.

* *p*<0.05

** *p*<0.01

*** *p*<0.001.

## Discussion

With the revision of the DSM-IV (American Psychiatric Association, 2000) to DSM-5 (American Psychiatric Association, 2013), the diagnostic criteria for PTSD have undergone several changes. Although there is great interest in how the new DSM-5 criteria will affect rates of PTSD, only very few studies to date have examined the impact of the introduced DSM-5 modifications to PTSD diagnosis (Elhai et al., 2012; Miller et al., 2013). The major goal of the present study was to compare rates of PTSD according to the DSM-5 with DSM-IV classification rules in a sample of ex-combatants from the DRC. Moreover, we aimed to investigate whether PTSD symptom severity is associated with perpetrated violent acts while controlling for general traumatic events.

All ex-combatants in the present study reported exposure to at least one potentially traumatic event. We found that the changes associated with DSM-5 resulted in an increase in the observed PTSD rate: 49.5% of the total sample met DSM-5 diagnostic criteria for PTSD, whereas only 44.2% met the diagnostic criteria according to the DSM-IV algorithm, and 41.0% fulfilled both sets of criteria. Recent studies with former combatants in eastern DRC reported a lower PTSD rate of 21% (Hecker, Hermenau, Maedl, Schauer, & Elbert, 2013). The increasing violence in the eastern DRC several months before the present study was conducted might have contributed to this elevated PTSD rate. Other authors have not consistently reported that the changes associated with DSM-5 might result in increased rates in diagnosed PTSD. Elhai et al. (2012) reported a non-significantly elevated rate of current PTSD for the DSM-5 diagnostic algorithm (compared to the DSM-IV symptom criteria). Miller et al. (2013) found comparable PTSD rates when diagnosed by the DSM-IV or the DSM-5 diagnostic system among a nationally representative sample of American adults; in contrast, rates were higher according to DSM-IV compared to DSM-5 in a clinical convenience sample of US military veterans. According to Calhoun et al. (2012), the differences in observed prevalence rates greatly depend on the observed base-rate in a given sample. The authors propose that the DSM-5 algorithm would result in an increase in observed prevalence until a DSM-IV diagnosis of approximately 50%; however, after having exceeded the threshold of 50% DSM-IV diagnosis, the DSM-5 algorithm would result in a decrease in observed prevalence. In the present study, a large overlap between the two diagnostic algorithms was observed: of the ex-combatants with DSM-IV PTSD, 92.9% also met DSM-5 PTSD; of those who met criteria for a current DSM-5 diagnosis, 83.0% fulfilled DSM-IV PTSD diagnosis. This is in line with the findings of Miller et al. (2013), who found that 86% of veterans with DSM-5 PTSD also had DSM-IV PTSD. Results of the present study showed that in 11.6% of the cases, the two diagnostic algorithms produced a differential diagnostic outcome. More PTSD cases were produced according to DSM-5 than using DSM-IV, as more new diagnostic cases were produced (8.4%) than were omitted (3.2%). If the DSM-5 is set as the current “gold standard” then DSM-IV would have produced more false negatives than false positives.

Some researchers have noted that participation in intense violence against others may be considered as potentially traumatizing and may also cause symptoms of PTSD (MacNair, 2002; Staub, 2006). The results of the present study indicate that only a few perpetrators (18.9%) identified an event during which they had perpetrated violence as the most distressing event to which their PTSD symptomatology referred, indicating that perpetrating violence is perceived as traumatic for some combatants, but not for all. This finding aligns with previous studies that showed that only a minority of perpetrators indicated an event that they had perpetrated as their most frightening event (Schaal et al., 2014). However, in the present study, those ex-combatants who indicated a perpetrated violent act as their index trauma were significantly more affected by DSM-5 PTSD compared to those who reported an event from the traumatic event list. This implies that although few ex-combatants indicated a perpetrated event as their most stressful experience, those who did were particularly affected by PTSD.

As a second goal, we investigated the impact of the number of perpetrated violent acts on symptoms of PTSD. The hypothesis that, after accounting for the number of traumatic event types, the number of perpetrated violent acts would be associated with symptoms of PTSD could not be confirmed. The number of perpetrated violent acts did not contribute to PTSD symptom severity, beyond general lifetime traumatic events. In line with other research (e.g., Schaal & Elbert, 2006), the results of this study revealed that the number of traumatic event types (experienced and witnessed) positively correlated with PTSD symptom severity. The ex-combatants were traumatized by their repeated exposure to traumatic stressors rather than by the number of their perpetrated violent acts. One reason why participants did not become psychologically distressed by their perpetrated violent offenses may be the fascinating and excited perception of the violent acts (Konner, 2006; Maclure & Denov, 2006). This phenomenon has been called appetitive aggression and describes that violence itself may be positive and appealing for the perpetrators (Elbert, Weierstall, & Schauer, 2010). Thus it is an intrinsic reward which drives aggression and not the instrumental gain. Such a protective influence of appetitive aggression on PTSD symptom severity has already been reported in other studies (Hecker et al., 2013; Weierstall, Schaal, Schalinski, Dusingizemungu, & Elbert, 2011). According to Pollock (1999), the development of PTSD in homicide perpetrators depends on the form of violence used. Reactive, unpremeditated violence—not instrumental violence—was associated with a current diagnosis of PTSD in homicide perpetrators (Pollock, 1999). Previous studies have shown high levels of appetitive aggression in Congolese ex-combatants (Hecker et al., 2013). From the results of the present study, it can be concluded that the subjective perception of the perpetrated violence—rather than the number of perpetrated events—might be of importance for the development of PTSD. The current study has a number of limitations. Its findings cannot be generalized to all types of fighters as the interviewed participants had left their armed group and were enrolled in the demobilization program of the United Nations. However, we interviewed all ex-combatants who joined the camp during a given time period. Because of the cross-sectional and retrospective nature of the design, it is impossible to establish causal or temporal relationships between the different variables. The newly developed items referring to the DSM-5 symptom criteria were still untested and not validated at the time of the conduct of the study. Socially desirable responses can never be completely ruled out. However, the participation of respondents was anonymous and questioning took place independent of the camp in an explicit research context, thereby reducing the likelihood of a strong bias. Moreover, we had previously validated this type of structured interviewing using physiological markers such as cortisol released over the last month in East-African ex-combatants (Steudte et al., 2011). A positive association was found between hair cortisol levels and the number of lifetime traumatic events and between PTSD and hair cortisol concentrations (Steudte et al., 2011). Although these data indicate that PTSD in severely traumatized individuals might be associated with hypercortisolism, they also validate both the assessment of traumatic events and the PTSD diagnosis based on PDS. A methodological strength of the present study is that its results were based on structured clinical interviews and that the reported findings are based on a study that was conducted under the challenging conditions of an ongoing conflict zone.

In conclusion, the findings demonstrate an increase in DSM-5 PTSD rates compared to the DSM-IV system, as more new diagnostic cases were produced with the DSM-5 diagnostic rules than were dropped. The results emphasize the need for thorough diagnostic evaluations and evidence-based treatments of PTSD in Congolese ex-combatants. The restoration of the psychological functioning of former combatants might also facilitate their reintegration process. The success of reintegration programs can be blocked by mental health problems and may even cause discontinuation of such programs. After their drop-out many might consider voluntarily rejoining the armed groups. A recent study with former combatants in the DRC demonstrated that PTSD can be effectively treated in former child soldiers and ex-combatants (Hermenau, Hecker, Schaal, Maedl, & Elbert, 2013).

## Supplementary Material

Posttraumatic stress disorder according to DSM-5 and DSM-IV diagnostic criteria: a comparison in a sample of Congolese ex-combatants

Posttraumatic stress disorder according to DSM-5 and DSM-IV diagnostic criteria: a comparison in a sample of Congolese ex-combatants
