# Supplementary material for: Posttraumatic stress disorder according to DSM-5 and DSM-IV diagnostic criteria: a comparison in a sample of Congolese ex-combatants
Source: Eur J Psychotraumatol. 2015 Feb 25;6:10.3402/ejpt.v6.24981. doi: 10.3402/ejpt.v6.24981 (PMC4342502; doi:10.3402/ejpt.v6.24981)
Supplement: Posttraumatic stress disorder according to DSM-5 and DSM-IV diagnostic criteria: a comparison in a sample of Congolese ex-combatants [file EJPT-6-24981-s001.pdf]

Hintergrund: Die Diagnosekriterien für PTBS sind im DSM-5, im Vergleich zum DSM-IV, verändert worden.

Susanne Schaal, Anke Koebach, Harald Hinkel, Thomas Elbert

Ziel: Das erste Ziel dieser Studie war, die Auswirkung dieser Modifikation auf die PTBS-Raten in einer Stichprobe von kongolesischen ehemaligen Kämpfern zu untersuchen. Als zweites Ziel untersuchte die Studie, ob die PTBS-Symptome mit täterbezogenen Handlungen oder mit traumatischen Ereignissen, die als Opfer erlebt wurden, zusammenhängen.
Methode: Es wurden 95 männliche ehemalige Kämpfer aus dem Osten der Demokratischen Republik Kongo interviewt. Sowohl die DSM-IV als auch die DSM-5 Symptomkriterien der PTBS wurden erhoben.
Ergebnisse: Die DSM-5 Diagnosekriterien ergaben eine PTBS Rate von 50% (n = 47), während die DSM-IV Symptomkriterien von 44% (n = 42) erfüllt wurden. Wenn man das DSM-5 als derzeitigen „goldenen Standard“ setzen würde, hätte das DSM-IV mehr falsch Negative (8%) als falsch Positive (3%) produziert. Eine Minderheit der Teilnehmer (19%, n = 18) gab als stressvollstes Ereignis ein Erlebnis an, in dem sie als Täter involviert war. Ergebnisse der Regressionsanalyse (R^2 = 0.40) zeigten, dass unter Kontrolle der Anzahl der Arten der traumatischen Ereignisse, die ausgeübten Gewalttaten nicht mit der PTBS Symptomschwere zusammenhingen.
Schlussfolgerungen: Die Ergebnisse zeigen, dass auf Grundlage der diagnostischen Regeln des DSM-5 mehr diagnostische Fälle produziert wurden als weggefallen sind, was im Vergleich zum DSM-IV zu einer Zunahme der PTBS Raten führte. Der fehlende Zusammenhang zwischen PTBS Symptomen und ausgeübten Gewalttaten könnte mit der potentiellen faszinierenden und aufregenden Wahrnehmung dieser Handlungen erklärt werden.

Citation: European Journal of Psychotraumatology 2015, **6**: 24981 - http://dx.doi.org/10.3402/ejpt.v6.24981
