# Supplementary material for: Posttraumatic stress disorder according to DSM-5 and DSM-IV diagnostic criteria: a comparison in a sample of Congolese ex-combatants
Source: Eur J Psychotraumatol. 2015 Feb 25;6:10.3402/ejpt.v6.24981. doi: 10.3402/ejpt.v6.24981 (PMC4342502; doi:10.3402/ejpt.v6.24981)
Supplement: Posttraumatic stress disorder according to DSM-5 and DSM-IV diagnostic criteria: a comparison in a sample of Congolese ex-combatants [file EJPT-6-24981-s002.pdf]

Title: El trastorno de estrés postraumático según los criterios diagnósticos del DSM-5 y el DSM-IV: Comparativa de una muestra de ex-combatientes congoleños

Susanne Schaal

Abstract
Antecedentes: En comparación con el DSM-IV, los criterios para el diagnóstico de trastorno de estrés postraumático han sido modificados en el DSM-5.
Objetivo: El primer objetivo de este estudio fue examinar el impacto de estas modificaciones en las tasas de TEPT en una muestra de ex-combatientes congoleños. El segundo objetivo de este estudio fue investigar si los síntomas de TEPT se asociabann con actos relacionados con ser el agresor o con eventos traumáticos relacionados con ser la víctima.
Método: Se entrevistó a noventa y cinco hombres ex-combatientes en la República Democrática del Congo. Se evaluaron tanto los criterios de síntomas de TEPT del DSM-5 como del DSM-IV.
Resultados: Los criterios de los síntomas del DSM-5 resultaron en una tasa de TEPT del 50% (n = 47), mientras que los criterios de los síntomas del DSM-IV se cumplieron en un 44% (n = 42). Si se estableciera el DSM-5 como la actual "regla de oro", entonces, el DSM-IV habría producido más falsos negativos (8%) que falsos positivos (3%). Una minoría de los participantes (19%, n = 18) señalaron un evento en el que estaban involucrados como agresores como su evento más estresante. Los resultados de un análisis de regresión (R2 = 0.40) mostraron que, después de contar el número de tipos de eventos traumáticos, los actos violentos perpetrados no se asociaron con la gravedad de los síntomas del TEPT.
Conclusiones: Los resultados demuestran que se producían más diagnósticos de casos con las reglas de diagnóstico del DSM-5 de los que se descartaban, dando como resultado un aumento en las tasas de TEPT en comparación con el sistema del DSM-IV. La asociación que falta entre los síntomas de TEPT y los actos violentos perpetrados podría explicarse por una potencial percepción fascinante y excitante de estos actos .

Keywords: trastorno de estrés postraumático; DSM-5; DSM-IV; Congo; ex-combatientes; violencia

Name of translator: Miriam Ramos Morrison

Citation: European Journal of Psychotraumatology 2015, 6: 24981 - http://dx.doi.org/10.3402/ejpt.v6.24981
